# Ameliorative effect of taurine-chloramine in azathioprine-induced testicular damage; a deeper insight into the mechanism of protection

**DOI:** 10.1186/s12906-018-2272-z

**Published:** 2018-09-17

**Authors:** Mona F. Schaalan, Basma K. Ramadan, Azza H. Abd Elwahab

**Affiliations:** 1 Department of Clinical Pharmacy, Faculty of Pharmacy, Misr International University, Km 28, Cairo-Ismailia road, PO Box 1, Heliopolis, Cairo, Egypt; 2 Department of Physiology, Faculty of Medicine for Girls (Cairo), Al-Azhar University, Cairo, Egypt

**Keywords:** Taurine-chloramine, Testicular atrophy, Azathioprine, Bcl-2, Caspase-9, DNA fragmentation, IL-1β

## Abstract

**Background:**

The male reproductive system is a sensitive and intricate process that can be distressed following exposure to various toxicants. Therapeutic drugs, especially chemotherapeutics, can also adversely affect male fertility by instigating hormonal changes leading to testicular cells injury. Azathioprine (AZA) is an effective anticancer drug, but some cases of testicular toxicity have been reported. The aim of this work was to investigate the protective effects of taurine chloramine (TAU-Cl), a reported antioxidant and antiinflammtory peptide, against AZA-induced testicular dysfunction in male rats and ascertain the contributing mechanisms.

**Methods:**

Forty male rats were allocated into four equal groups; (i) normal control rats, (ii) TAU-Cl group (100 mg/kg b.w/day for 10 weeks, (iii) AZA group (5 mg/day for 4 weeks); (iv) TAU-Cl/AZA group.

**Results:**

AZA caused increased DNA damage in the testes, and alterations in sex hormones and sperm quality, including sperm count, viability, and motility. Moreover, testicular tissue from the AZA-treated group had increased levels of oxidative stress indicator, MDA, and decreased activity of the antioxidant enzymes as superoxide dismutase (SOD), reduced glutathione (GSH) and catalase (CAT) levels. These deleterious events were accompanied by upregulated levels of the pro-inflammatory cytokines, tumor necrosis factor-alpha (TNF-α), and protein expression of iNOS and NFκB-p65, interleukin-1beta (IL-1β), and proapoptotic marker; caspase-9, together with decreased Bcl-2, NrF2 and hemeoxygenase (HO-1) expression. In contrast, TAU-Cl pretreatment significantly abrogated these toxic effects which were confirmed histologically.

**Conclusion:**

Pretreatment with TAU-Cl exerts a protective effect against AZA-induced male reproductive testicular atrophy. This finding could open new avenues for the use of TAU-Cl as a complementary approach to chemotherapy supportive care.

## Background

Azathioprine (AZA) is an immunomodulatory and cytotoxic drug often used to treat inflammatory bowel disease, autoimmune disorders, organ transplant rejection, and cancer [1]. It functions via multifaceted pathways; it inhibits purine metabolism, which leads to DNA damage [2], and at high chemotherapeutic doses inhibits DNA synthesis. By contrast, its anti-inflammatory effect is mainly mediated via inhibition of the small GTPase Rac1, leading to apoptosis of activated T-lymphocytes [3]. AZA also increases oxidative stress; upon its administration, as it is rapidly metabolized into several toxic and non-toxic metabolic compounds, including the active 6-mercaptopurine (6-MP) that is formed through a conjugation reaction with glutathione (GSH). This leads to the depletion of GSH [4] and a surge in reactive oxygen species (ROS). 6-MP is further metabolized by xanthine oxidase (XO) to thiouric acid, and this reaction also creates ROS [5].

Despite their effectiveness, antimetabolic drugs may cause drug-induced toxicity with increased risk of death, even when used at standard doses [6]. As expected for immunosuppressive drugs, common side effects of AZA treatment in both animals and humans are bone marrow suppression and lymphocyte depletion. However, its active metabolite 6-mercaptopurine (6-MP) damages rapidly dividing cells, such as those in the bone marrow, intestinal epithelium, and reproductive organs of adults [7, 8]. One of these major drug–related disorders is testicular atrophy and infertility, with genetic polymorphisms of the thiopurine methyltransferase enzyme, which is responsible for thiopurine metabolism, possibly contributing to its mechanism. Population studies have shown that patients with low enzymatic activity have a high risk for severe, potentially fatal toxicities [9].

The usage of nutraceuticals, amino acids and vitamins as adjunctive therapy to chemotherapeutic agents and drugs with reported toxicity is effective in improving drug safety and reducing toxicities and side effects [10]. One promising nutraceutical that is commonly used as an adjuvant for chemotherapy is taurine (TAU), which is a sulfur-containing amino acid that does not contribute to protein synthesis and that is traditionally considered as an inert molecule without any reactive groups. It can be obtained either exogenously through dietary source as poultry, beef, pork, seafood, and processed meats or endogenously through biosynthesis from methionine and cysteine precursors. Both sources are important to maintain the physiologic levels of taurine, and either can help to compensate the other in cases of deficiency. Taurine supplementation has been proposed to have beneficial effects in the treatment of epilepsy, heart failure and cystic fibrosis [11].

Interestingly, TAU has been detected in the testes of humans and has been identified as the major free amino acid of sperm cells and seminal fluid [12]. It has been localized to Leydig cells of the testes, the cellular source of testosterone in males, and to the cremaster muscle, efferent ducts, and peritubular myoid cells surrounding seminiferous tubules [13]. The major function of taurine in leukocytes, neutophile and any inflammed cell is to trap chlorinated oxidants (HOCl) and convert them into less toxic taurine chloramine (TAU-Cl) and TAU-Cl production is found to result in decreased NO production [14].

It has become increasingly apparent that oxidative stress plays a major role in a broad range of human diseases and in many diseases including the destruction of male rat reproductive system. By virtue of its antioxidant activity, TAU-Cl also plays a crucial role as a cytoprotectant and attenuates apoptosis in several inflammatory and chronic diseases [15]. There is growing consensus that the beneficial effects of TAU-Cl are due to its antioxidant properties, aided by its ability to improve mitochondrial function by stabilizing the electron transport chain and inhibiting ROS generation [16].

Taurine levels in spermatozoa are also correlated with sperm quality, presumably due to its antioxidant activity that protects against lipid peroxidation and its effect on spermatozoa maturation by facilitating the capacitation, motility, and acrosomal reaction of sperm [17, 18]. To this end, this study aimed to elucidate the toxic effect of AZA on the testicular functions in male rats and unravel the potential protective effects of TAU-Cl combination with AZA, focusing on the induced inflammation, oxidative perturbations and apoptosis.

## Material and methods

### Material

Commercial chow diet (balanced diet), containing 67% carbohydrates, 10% fat, and 23% protein as the energy sources (overall calories: 3.6 kcal/g), was purchased from El Gomhorya company (Cairo, Egypt). Azathioprine (AZA): manufactured by EXCELLA Gmbh & Co. Feucht, Germany. Taurine (TAU): 2-amino ethane sulfonic acid was supplied by GALL Pharma, Austria Pharmaceutical. Taurine-chloramine (TAU-Cl), N-Monochlorotaurine, was synthesized freshly on the day of use by adding equimolar amounts of NaOCl (Sigma-Aldrich) to taurine. This was prepared by dropwise additions of 5 ml of 2 mM NaOC1 (Sigma) solution in 0.06 M phosphate buffer (pH 7.4-7.5) with vigorous stirring to 5 ml of 29 mM amine solution in the same buffer. The authenticity of TAU-Cl formation was monitored by UV absorption (200–400 nm). Its sodium salt form (molecular weight 181.57) was prepared, and its purity was checked according to a previously published method of Gottardi and Nagl (2002) [14]. Stock solution of taurine chloramine was kept at 4 ºC for a maximum period of 2 weeks before use.

## Methods

### Experimental design

Adult male albino rats (Nile Pharmaceuticals Company, Cairo, Egypt), 7-8-weeks old weighing 130-150 g, were housed in laboratory standard cages, under a thermostatically regulated, light controlled condition with 12 h-light and -dark cycles. Animals were acclimatized for one week before the initiation of the study in the laboratory of Physiology, Faculty of Medicine AI-Azhar University, and were kept on standard laboratory chow and water ad libitum. Experimental design and animal handling were according to the guidelines and ethical procedures and policies approved by Animal Care and Use Committee of Faculty of Medicine, AI-Azhar University, Cairo, Egypt.

Forty rats were randomly assigned into 4 groups (*n*=10), where in group I, rats received normal balanced chow and saline orally by gastric gavage tube for 10 weeks to serve as normal control group. The second group of rats (TAU-CL), received TAU-CL daily at a dose of 100 mg/kg b.w. orally, which was freshly prepared before use for 10 weeks. Animals in group III (AZA) were treated with AZA (5 mg/kg b.w. in distilled water, po) for four weeks after six weeks of normal diet. TAU-CL (100 mg/kg b.w. p.o) was administered for 6 weeks in rats of group IV (TAU-CL/AZA), and continued with AZA (5 mg/kg b.w.) for four weeks.

### Serum and tissue collection

All four groups continued their indicated diet till the end of experiment and the last dose of any treatment was given 24 hours before killing the rats, At the time of carnage, animals were weighed and blood was collected from retro-orbital venous plexus by capillary tubes under light phenobarbitone anesthesia, and centrifuged (3000g, 4 ºC, 20 min) to separate sera. Sera were separated in aliquots in Eppendorf tubes and stored frozen at -80°C until analysis for detection of sex hormone activities. Finally, the animals were euthanized by cervical dislocation (physical method of euthanasia of small animals by applying pressure to the neck and dislocating the spinal column from the skull or brain). The aim is to quickly separate the spinal cord from the brain so as to provide the animal with a fast and painless death. After that, both testes were removed, the right one was used for preparation of the homogenate for further investigation of tissue sample molecular investigation , oxidative stress marker activities, pro-inflammatory cytokines, apoptotic assay and expression of iNOS and NFκB-p65 using Western blotting, and Nrf2 and HO-1 by Quantitative Real-Time PCR in the testis. The left testis was rapidly immersed in Bouin's fixative for 24 hr for histology and immunohistochemistry [19].

#### Semen evaluation

Cauda epididymis were used to study sperm abnormalities including; *sperm motility, epididymal count and vitality.* They were held in 4 ml of saline solution (0.9% NaCl); by squeezing and subsequent homogenization process, the sperms became free in the saline solution. A haemocytometer slide was used for sperm counting; the sperms were counted in four squares at 40 magnifications. The motility assessment was expressed as percentage motile forms. The epididymal filtrate was then mixed in equal volume with eosin-nigrosin stain and a smear made of it was used for epididymal sperm vitality [20]. The caudal epididymal sperm reserve was determined using standard hemocytometric method [21].

##### Tissue preparation of the homogenate

The right testis was washed in ice-cold saline and kept in 1 ml cold physiological saline (0.9% NaCl). Each testis was sliced into two parts; the first was homogenized in 1 ml physiological saline and centrifuged at 20,000×*g* for 30 min at 4 °C. The supernatants were collected and stored at − 20 °C for the assessment of DNA fragmentation, MDA, GSH, NO, SOD, CAT, IL-1β, TNF-α. For western blotting of BCL-2, iNOS and NFκB-p65, the other part of the right testis is homogenized in RIPA buffer (1% NP-40, 0.5% sodium deoxycholate, 1% SDS in PBS with protease inhibitors, pH *8.0*) supplemented with protease inhibitors using the liquid nitrogen grinding, followed by incubation on ice for 10 min. The samples were centrifuged thoroughly (10,000 g for 10 min at 4 °C) to obtain protein supernatants.

### Biochemical analysis

#### Sex hormone assay

Serum levels of testosterone, Luteinizing hormone (LH) and Follicle stimulating hormone (FSH), were estimated using enzyme-linked immunosorbent assay (ELISA) kits (Diagnostic System Laboratories Inc., USA), according to the manufacturer’s instruction.

The detection limits of the assay were 0.2 mIU/mL for LH and 0.1 mIU/mL for FSH. Intra- and inter-assay coefficients of variation (CV) were 3.2 and 6.7% for LH, and 3.3 and 7.1% for FSH, respectively. For total testosterone, the assay sensitivity, 0.066 ng/ml; intra-assay and interassay coefficients of variation, 6.5–11.1 and 9.3–11.3%, respectively; accuracy, 84–123%).

### Molecular investigation (DNA fragmentation assay)

Nucleic acid extraction was done according to the chemical method of Collins et al. (1997) [22] with Diphenylamine using Agaros gel electrophoresis analysis. After tissue homogenization, centrifugation and precipitation of DNA fragmentation was done by addition of 1 ml Triton-X buffer (pH;7.4) and vortexing vigorously to allow the release of fragmented DNA. DNA was hydrolyzed by adding 160 μl of 5% Trichloroacetic acid (TCA) to each pellet and heating 15 min at 90° C in a heating block. Colorimetrical quantitation on staining with diphenylamine (DPA) was assessed at a wave length 600 nm against blank reagent and the values are given as % fragmented DNA.

### Assessment of oxidant/antioxidant status in testicular tissues

The testicular content of lipid peroxides was quantified as MDA according to the method described by Ohkawa et al. (1979) [23]. Reduced GSH was measured using the method described by Jollow et al. (1974) [24]. SOD activity in testicular cells was estimated following the method described by Misra (1989) [25]. Catalase (CAT) activity was estimated using hydrogen peroxide as substrate according to the method of Clairborne (1995) [26].

### Assay of inflammation and apoptosis in testicular homogenate

The extent of inflammation in testis samples was estimated by measuring IL-1*β* and TNF- *α* levels using commercial ELISA kits according to the manufacturer’s instructions (R & D systems, USA). Apoptosis was assayed by measuring the activity of caspase-9 using Caspase-Glo-9 assay kit according to the manufacturer’s instructions (Promega, Wisconsin, USA).

#### Western blotting assays

Testicles samples were homogenized in ice-cold lysis buffer, and the homogenates were centrifuged at 14,000×g for 20 min at 4 °C. Samples’ protein contents were determined according to the method of Bradford (Bio-Rad Laboratories, Watford, UK) [27]. Samples of equal protein concentrations were electrophoresed using 10% SDS/PAGE and electro-transferred to polyvinylidene difluoride membranes. The membranes were blocked with 5% (*w*/*v*) skimmed milk powder in PBS/Tween-20 for 2 h at room temperature. Then, the membranes were incubated with anti-Bcl2 (Santa Cruz Biotechnology), anti-iNOS (Santa Cruz Biotechnology), and anti-NFκB-p65 antibodies (1:250) diluted in tris-buffered saline-tween containing 1% bovine serum albumin, and β actin (Santa Cruz Biotechnology) as internal control diluted 1:1000 in blocking buffer. The membranes were incubated with the corresponding secondary antibodies for 1 h at room temperature, washed, and then developed. Finally, images of indicated protein bands were recorded on the BioMax film (Kodak), and densitometrcally quantification was conducted by using Image J software (Bio-Rad, California, USA). Densities of bands were standardized to the corresponding density of β-actin.

### Quantitative real-time PCR

RNA in the testis samples was isolated utilizing the TRIzol reagent (Invitrogen, CA, USA), and 1 μg of the isolated RNA was used as a template together with random primers to synthesize cDNA utilizing Thermo Scientific Maxima First Strand cDNA Synthesis Kit for RT-qPCR. Each cDNA sample was run in triplicate for real-time PCR analysis. GAPDH (accession number: NM_017008.4; sense:5-GCATCTTCTTGTGCAGTGCC 3; antisense: 5-GATGGTGATG GGTTTCCCGT-3) served as a housekeeping gene. Real-time PCR reactions were performed utilizing the Power SYBR Green Applied Biosystems 7500 System (Life Technologies, CA, USA) at 94 °C for 4 min, followed by 42 cycles at 94 °C for1 min, at 60°Cfor1 min, and then held for the final phase at 72 °C for 10 min. Gene expression analysis employed the 2 − ΔΔCt method according to Pfaffl [28]. The PCR primers for the following genes were synthesized by In vitrogen:

**Table:** 

-Nrf2 (accession number: NM_031789.2;	sense: 5’-GGTTGCCCACATTCCCAAAC- 3′
	antisense: 5’-GGCTGGGAATATCCAGGGC-3′
-HO-1 (accession number: NM_012580.2;	sense: 5’-GCGAAACAAGCAGAACCCA-3′
	antisense: 5’-GCTCAGGATGAGTACCTCCC-3′

### Histopathological analysis

#### Light microscopic examination

After removal of the left testis, it was weighed and rapidly immersed in Bouin’s fixative for 24 h, then, washed in several changes of 70% ethanol, dehydrated, cleared and embedded in paraffin. The tissue was sectioned at 5 μm thick, mounted and stained with Hematoxylin and Eosin (H&E) for studying the histological architecture of testis.

#### Statistical analysis

All the data were expressed as mean ± standard error (SEM). Statistical analysis was performed using one-way analysis of variance (ANOVA) followed by Bonferroni post hoc multiple comparison test using the program Statistical Package for the Social Sciences (SPSS). The values of *P* < 0.05 were considered significant.

## Results

### Effect of AZA and TAU-Cl on rat body and testis weights, and on levels of serum testosterone, LH and FSH

Since there was no significant difference across any parameters examined between the control and TAU-Cl groups, the comparison was referred to the control group.

We first investigated the effects of AZA alone and with TAU-Cl pre-treatment on the overall body weight and testis weights of male rats. As shown in Table 1, there was a significant decrease in body and testis weights in the AZA-treated group (8.7 and 22.4%, respectively, at *P* < 0.05) compared to the control group. TAU-CL pre-treatment successfully normalized the body and testis weight compared to the AZA-treated group.

**Table 1:** Effects of TAU-CL, AZA, and their combination on body and testicular weight

Groups	Group I (Control)	Group II (TAU-CL)	Group III (AZA)	Group IV (TAU-CL/AZA)
Parameters				
Body weight (g)	285 ± 2.52	289 ± 2.92	260^a^ ± 1.59	275 ^a,b^ ± 4.32
Testicular weight (g)	1.25 ± 0.52	1.85 ± 0.43	0.97^a^ ± 0.33	1.19^b^ ± 0.72

Assessment of body and testicular weight in the experimental groups, Control, TAU-CL, AZA, TAU-CL/AZA. Data are expressed as the mean ± SEM, and data were analyzed using one-way ANOVA followed by the Bonferroni post hoc multiple comparison test (*n* = 10). Difference between groups were considered statistically significant at *P* ≤ 0.05. (a) Significant values versus the group control, (b) Significant values versus the AZA group

*TAU-CL* rats treated with taurine, *AZA* rats given Azathioprine, *TAU-CL/AZA* Rats given azathioprine and treated with taurine-chloramine

**Fig. 1:**
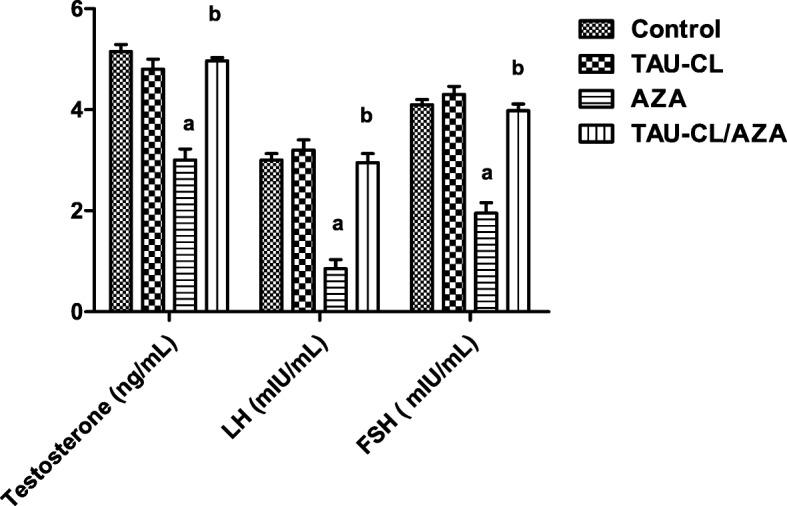
Effects of TAU-CL, AZA, and their combination on serum sex hormone levels, testosterone, Luteinizing hormone (LH) and Follicle stimulating hormone (FSH). Assessment of serum sex hormone levels, testosterone, Luteinizing hormone (LH) and Follicle stimulating hormone (FSH) in the experimental groups, Control, TAU-CL, AZA, TAU-CL/AZA. Data are expressed as the mean ± SEM, and data were analyzed using one-way ANOVA followed by the Bonferroni post hoc multiple comparison test (*n* = 10). Difference between groups were considered statistically significant at P ≤ 0.05. **a** Significant values versus the group control, (**b**) Significant values versus the AZA group

Next, we looked at the levels of sex hormones in each of the treatment groups (Fig. 1). AZA had a negative effect on steroidogenic hormones, reflected by significant decreases in the serum levels of testosterone (41.74%), LH (71.6%), and FSH (52.4%) compared to control. With TAU-CL pre-treatment, the altered levels returned to near normal.

In summary, AZA has a significant effect on testicular size and sex hormones, as expected, and TAU-CL pre-treatment appears to protect against this damage. Since there was no difference in body weight, testis weight, or sex hormone levels between the TAU-CL-treated group and the control group, only the control group was used for statistical comparisons for this and subsequent experiments.

### Effect of AZA and TAU-CL on the testicular levels of pro-inflammatory cytokines and oxidative stress/antioxidant markers in rats

**Fig. 2:**
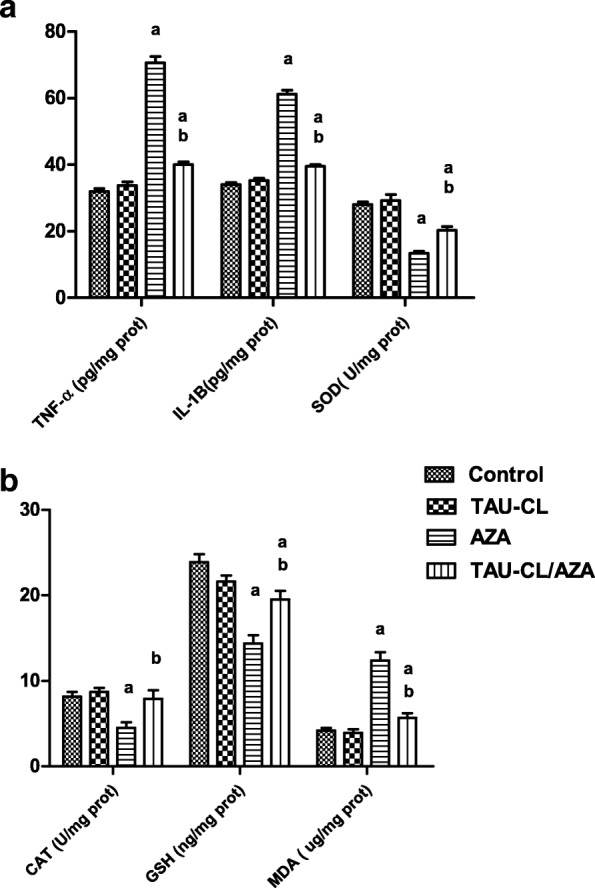
Effects of TAU-CL, AZA, and their combination on testicular levels of tumor necrosis factor alpha (TNF-α), interleukin 1-beta (IL-1β) and Superoxide dismutase (SOD) (**a**), catalase (CAT), glutathione (GSH) and malondialdhyde (MDA) (**b**) in experimental groups. Data are presented as the mean ± SEM and analyzed using one-way ANOVA followed by the Bonferroni post hoc multiple comparison test (n = 10). Differences between groups were considered statistically significant at P ≤ 0.05. **a** Significant versus the control group. **b** Significant versus the AZA group

Assessment of the testicular levels of pro-inflammatory cytokines tumor necrosis factor alpha (TNF-α) and interleukin 1 beta (IL-1β) were significantly increased in AZA-treated rats (121.3 and 80%, respectively, *P* < 0.05) compared to control animals (Fig. 2a). TAU-CL supplementation for 10 weeks successfully ameliorated the elevated cytokine levels compared to AZA treatment.

AZA supplementation for 4 weeks induced considerable oxidative stress, reflected by a significant increase in testicular malondialdehyde (MDA) levels (195.2%) and reductions in testicular superoxide dismutase (SOD) and catalase (CAT) activity (52.1 and 45.1%, respectively) and GSH levels (40%) compared to control (Fig. 2a,b). TAU-CL supplementation significantly corrected these changes induced by AZA treatment, and levels approached those in the control group.

#### Effect of AZA and TAU-CL on rat’s epididymal sperm count, motility and viability (live: Dead ratio) in AZA treated rats

Significant reductions in sperm motility (42.9%), caudal epididymal sperm count (67.6%), and viability (57.8%) were observed in AZA-treated rats compared to control rats (Table 2). These reductions were improved to near normal by TAU-CL pre-treatment of AZA-treated rats.

**Table 2:** Effects of TAU-CL, AZA, and their combination on percentage of sperm motility, epididymal count (10^6^/ml) and sperm vitality (Live: Death ratio)

Groups	Group I (Control)	Group II (TAU-CL)	Group III (AZA)	Group IV (TAU-CL/AZA)
Parameters				
Sperm motility (%)	90.45 ± 5.01	88.5 ± 4.51	51.59^a^ ± 1.59	86.13^b^ ± 4.32
Caudal epididymal sperm count (10^6^/ml)	95.01 ± 2.15	92.3 ± 2.21	30.75^a^ ± 2.66	89.99^b^ ± 3.65
Sperm vitality (live: dead ratio)	14.15 ± 1.02	13.8 ± 1.31	5.95^a^ ± 0.65	13.01^b^ ± 1.25

Assessment of percentage of sperm motility, epididymal count (10^6^/ml) and sperm vitality (Live: Death ratio) in the experimental groups; Control, TAU-CL, AZA, TAU-CL/AZA. Data are presented as the mean ± SEM, and data were analyzed using one-way ANOVA followed by the Bonferroni post hoc multiple comparison test (*n* = 10). Differences between groups were considered statistically significant at P ≤ 0.05. (a) Significant versus the control group. (b) Significant versus the AZA group

#### Histomorphometrical results

**Fig. 3:**
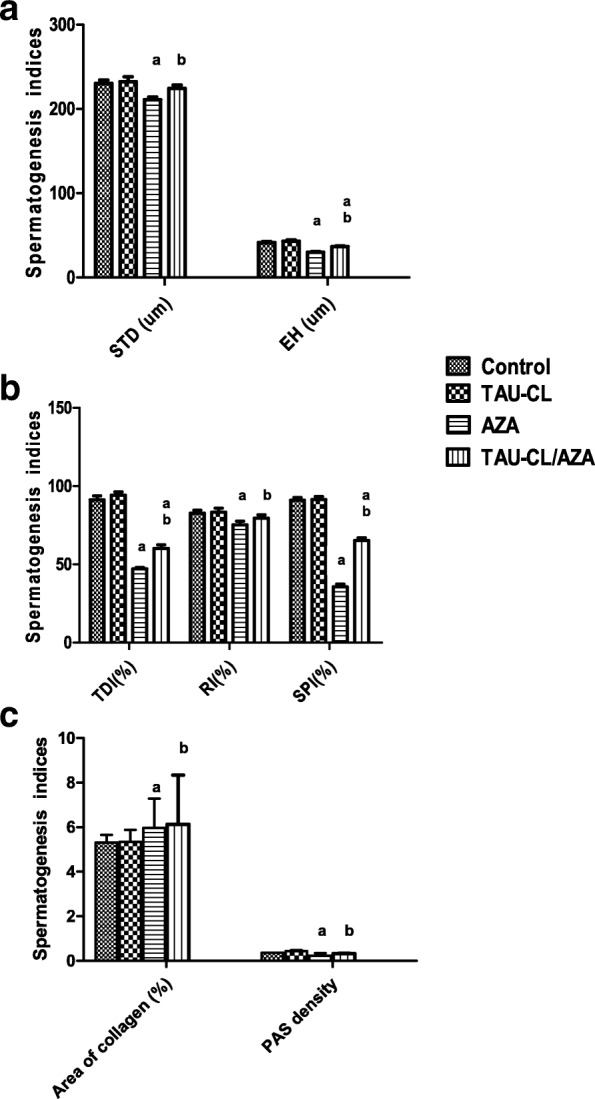
Effects of TAU-CL, AZA, and their combination on spermatogenesis indices in the seminiferous tubules; STD, EH, TDI %, RI%, SPI%, PAS optical density, Collagen fiber area (%). **a**; Seminiferous tubule diameter (STD) and Epithelial height (EH), **b**; Tubular differentiation index **(**TDI %), Repopulation index (RI%) and Spermiogenesis index (SPI%), and **c**; Area of collagen fiber (%) and Periodic acid Schiff reaction (PAS) optical density in the experimental groups; Control, TAU-CL, AZA, TAU-CL/AZA. Data are presented as the mean ± SEM, and data were analyzed using one-way ANOVA followed by the Bonferroni post hoc multiple comparison test (n = 10). Differences between groups were considered statistically significant at P ≤ 0.05. **a** Significant values versus group control, (**b**) Significant values versus AZA group. STD: Seminiferous tubule diameter, EH: Epithelial height, TDI: Tubular differentiation index, RI: Repopulation index, SPI: Spermiogenesis index, PAS: Periodic acid Schiff reaction

As presented in Fig. 3 panel, significant decreases in the seminiferous tubule diameter (STD), epithelial height (EH) (Fig. 3a), as well as tubular differentiation index (TDI), repopulation index (RI), spermiogenesis index (SPI)( Fig. 3b) added to PAS density (Fig. 3c) in the AZP group were noted. While a non-significant increase in the collagen fiber area percentage (Fig. 3c) was observed in the AZP group compared to the control group in Masson’s trichrome staining. These changes were significantly improved in the TAU/AZP-treated group compared to the group treated with AZP alone.

#### Effect of AZA and TAU-CL on DNA fragmentation and the expression of caspase-9 and Bcl-2

As evident in (Table 3), we observed a significant increase in DNA fragmentation from 70.55 ± 6 to 91.27 ± 10.5 after AZA treatment. Administration of TAU-CL prior to AZA treatment significantly decreased DNA fragmentation to 68.19 ± 4.8 compared to AZA treatment alone.

**Table 3:** Effects of TAU-CL, AZA, and their combination on the percentage of DNA fragmentation, Caspase 9 activity, and Bcl-2 expression in different groups

Groups	Group I (Control)	Group II (TAU-CL)	Group III (AZA)	Group IV (TAU-CL/AZA)
Parameters				
DNA fragmentation (%)	68.2 ± 5.01	70.55± 6	91.59^a^ ± 10.59	68.19^b^ ± 4.8
Caspase-9 activity (ng/g tissue)	1.6 ± 0.15	1.7 ± 0.07	2.5^a^ ± 0.13	1.6^b^ ± 0.17
BCL2 (ng/mg tissue)	1.25 ± 0.02	1.3 ± 0.08	0.75^a^ ± 0.05	1.2^b^ ± 0.13

The percentage of DNA fragmentation, Caspase 9 activity, and Bcl-2 expression, measured by western blot, β-actin was used as a loading control (C), in different groups

Data are expressed as the mean ± SEM, and data were analyzed using one-way ANOVA followed by the Bonferroni post hoc multiple comparison test (*n* = 10). Difference between groups were considered statistically significant at *P* ≤ 0.05. (a) Significant values versus the group control, (b) Significant values versus the AZA group

**Fig. 4:**
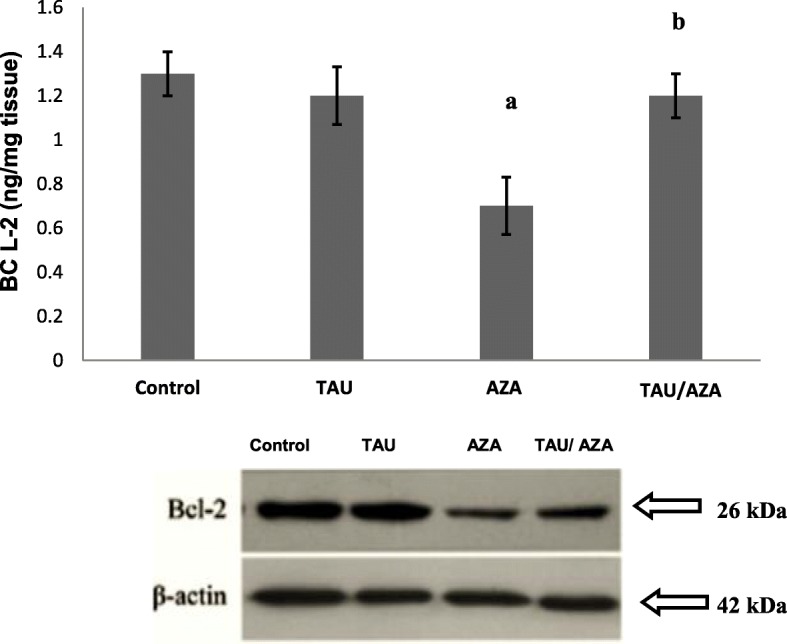
Effects of TAU, AZA, and their combination on Bcl-2 expression measured by western blot, β-actin was used as a loading control (**C**). Data are presented as the mean ± SEM (*n* = 10). (**a**) significant difference compared to the control group, (**b**) significant difference compared to the AZA group, all at *P* < 0.05

In AZA-treated rat testes homogenate, overall caspase-9 levels were significantly elevated from 1.7 ± 0.07 to 2.5 ± 0.13, while Bcl-2 levels were significantly reduced from 1.3 ± 0.08 to 0.7 ± 0.09 compared to control rats (Fig. 4). TAU-CL pre-treatment significantly increased Bcl-2 levels from 0.7 ± 0.09 to 1.2 ± 0.13 and significantly reduced caspase-9 levels from 2.5 ± 0.13 to 1.6 ± 0.1 compared to rats treated with AZA alone. Collectively, these results show that the Bcl-2-caspase-9 pathway is activated.

#### INOS and NF-κB-p65 expressions in testicular homogenate

**Fig. 5:**
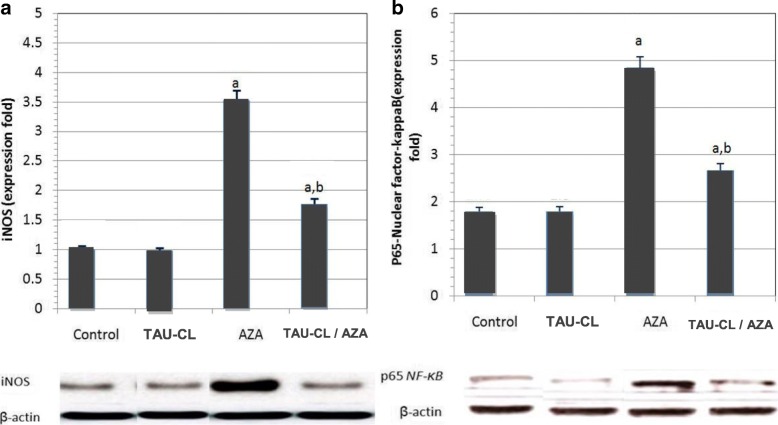
Western blotting analysis of testicular inducible nitric oxide synthase (**a**; iNOS) and p65 nuclear factor-kappa B (**b**; NF-ƘB p65) in Control, Taurine-chloramine (TAU-CL), Azathioprine (AZA) and TAU-CL/AZA groups. Data are presented as mean ± SEM, ^a^Significant change from the control group at *p* < 0.05; ^b^significant change from the AZA group at *p* < 0 05 using Tukey’s post hoc test

The testicular iNOS protein expressions were significantly higher (*p* < 0.05) in the AZA group in comparison with the control group, as presented in Fig. 5a. The TAU-CL-pretreatment and then concomitantly with AZA (group IV) showed significantly decreased (*p* < 0.05) iNOS protein expressions but not returned to the control group level. Testicular p65NF-κB expression was more activated in the AZA-treated groups. Oral supplementation of TAU-CL concomitantly with AZA treated rats caused a significant reduction (*p* < 0.05) of NF-κB-p65 expression (Fig. 5b).

#### Nrf2 and HO-1 overexpression protects against AZA-induced testicular injury

**Fig. 6:**
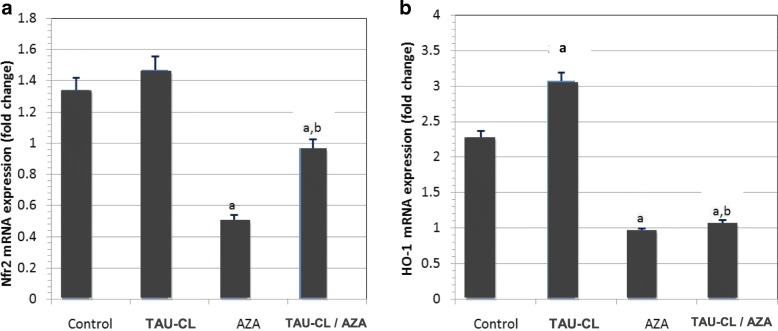
Effect of taurine-chloramine (TAU-CL) treatment on nuclear factor erythroid 2-related factor (**a**; Nrf2) and heme oxygenase-1 (**b**; HO-1) mRNA expression in the testis of rats treated with azathioprine (AZA). Data of the mRNA expression are expressed as the mean ± SEM of triplicate assays, normalized to the *GAPDH* mRNA level, and shown as fold change (in log2 scale) relative to the control mRNA levels. ^a^Significant change from the control group at *p* < 0 05; ^b^significant change from the AZA group at *p* < 0 05 using Tukey’s post hoc test

The mRNA expression levels of Nrf2 and HO-1 were significantly (p < 0 05) lower in the AZA-treated group than in the control group (Fig. 6a,b). Both Nrf2 and HO-1 mRNA expression were significantly upregulated in the TAU-CL/AZA group compared to the AZA; moreover, HO-1 mRNA level showed insignificant change (*p* > 0 05) compared to the control group; but Nrf2 mRNA expression in the TAU-CL/AZA group still significantly higher than in the control group.

#### Histopathology results

##### Light microscopy examination: (Fig. 7)

Histological examination of H&E-stained sections of rat testis from the control group (a-b) showed that the testis is formed by seminiferous tubules (STs) which are lined by spermatogenic cells and Sertoli cells. Leydig (interstitial) cells were present in the interstitial space between STs and surrounded by blood vessels as seen by H&E stain.

**Fig. 7:**
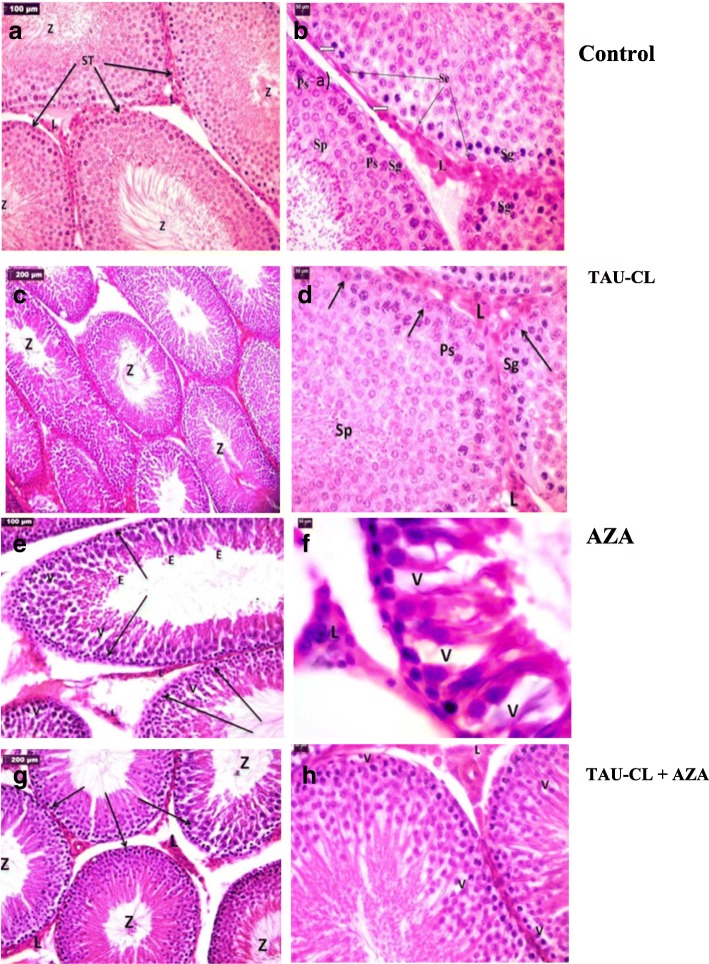
Photomicrographs of testis sections from all experimental groups. Light micrographs of testicular tissue sections from all experimental groups. Control group: (**a**) the normal pattern of seminiferous tubules (STs) full of spermatozoa (Z) with clusters of Leydig cells (L) between the tubules. **b**: STs lined by spermatogenic cells, including spermatogonia (Sg), primary spermatocytes (Ps), spermatids (Sp), spermatozoa (Z), and Sertoli cells (Se) (black arrows). Surrounded by myoid cells (white arrows) with clusters of Leydig cells in-between (L). TAU-CL group (**c**-**d**): the normal pattern of STs appears as the control. AZP group: (**e**) widely separated STs, some showing loss of normal architecture, with the presence of many vacuolated cells (v) and disorganized spermatogenic cells. **f**: the lumen contains some exfoliated, degenerate spermatogenic cells (**e**). TAU-CL & AZP group (**g**-**h**): showing a potentially preserved testicular architecture compared to control group

TAU-CL group (c-d): showing normal pattern of STs appears as the control.

Many significant histopathological changes were observed in the testis of rats in the AZA group (e-f), including widening of interstitial spaces, disruption and atrophy of the STs, disorganization of the spermatogonia, few spermatogenic cells and spermatozoa, numerous cells with pyknotic nuclei and eosinophilic or vacuolated cytoplasm. The majority of the affected germ cells were spermatogonia and spermatocytes. Some Leydig cells appeared atrophied with pyknotic nuclei. No obvious changes in collagen fibers in-between STs could be detected with Masson’s trichrome stain.

In the TAU-CL/AZA-treated group (g-h), testicular architecture appeared more or less similar to that of the control, with ameliorated changes, in spite of presence of some vacuolated cells.

## Discussion

The clinical use of AZA as an immunosuppressant and chemotherapeutic agent has been associated with various organ toxicities, however, reports on its safety regarding testicular functions are scarce. The current study highlights the gonadotoxicity of AZA, accompanied by the induction of inflammation, oxidative stress and apoptosis, and the efficacy of TAU-CL in providing testicular protection.

Spermatogenesis is highly susceptible to testicular inflammation, which causes damage to the seminiferous epithelium and increases apoptosis of spermatogenic cells. The effect of inflammation on spermatogenesis is evidenced by the histological results in this study, suggesting a direct association between AZA treatment and testicular inflammation, as shown by elevated testicular IL1-ß and TNF-α levels. This association is supported by the study of Ramonda et al. (2014) [29], who reported an increase in semen TNF-α levels associated with reduced sperm count, reduced motility, and altered morphology. These disturbances were successfully abrogated by TAU, as shown by Ahmed (2015) [30], who attributed these corrective effects to the anti-inflammatory, anti-apoptotic (through the intrinsic apoptosis pathway), and steroidogenic effects of TAU. The anti-inflammatory action of TAU-CL, evidenced in the current study, confirms the previous findings of Latchoumycandane et al. (2015) [31], who reported that TAU is effective as an anti-inflammatory supplement when given to alleviate the inflammation induced in the kidney by chronic ethanol ingestion.

Increased inflammation is associated with increased oxidative stress, which itself impairs sperm function [32]. Indeed, inflammatory damage to the male genital tract leads to the increased generation of ROS. Superoxide, hydroxyl, and hydrogen hydroxide radicals are the major ROS present in seminal plasma [33], and we chose to study the levels of the main free radical scavengers- SOD,CAT, and GST in this study.

We found that AZA treatment for 4 weeks significantly increased MDA levels and reduced GSH, SOD, and CAT levels; these decreases were corrected by TAU-CL pre-treatment. There is a positive correlation between abnormal and immature sperm and oxidative stress [34]. Consistent with these findings, we showed that the percentage of abnormal, immature, immotile, and dead sperm was significantly increased in the AZA group, where levels of SOD and CAT were reduced [35]. As ROS are known to promote apoptosis [36], it could be that this is the mechanism resulting in decreased sperm count and viability.

In keeping with the reported ability of TAU to scavenge ROS, and attenuate lipid peroxidation [15, 37], we found that pre-treatment with TAU-CL protected against the effects of AZA: restoring levels of oxidative stress markers and free radical scavengers to normal levels, and preventing the damage caused to sperm count and motility. The antioxidant effects observed with TAU-CL pre-treatment in this study may be associated with its sulfur moiety, and the modulation of GSH, SOD, and GSH levels by TAU is critical in the cellular defense against oxidative stress. As reported by Kim and Cha (2014) [15], taurine undergoes halogenation in phagocytes upon inflammation, and is converted to taurine bromamine and taurine chloramine (TAU-CL). The latter is released from activated apoptotic neutrophils and suppresses inflammatory mediators such as, superoxide anion, NO, TNF-α, and interleukins, and prostaglandins in inflammatory cells. Moreover, TAU-CL stimulates the expressions of antioxidant proteins (heme oxygenase 1, peroxiredoxin, thioredoxin), and enzymes (glutathione peroxidase, and catalase) in macrophages. Therefore, a vital anti-inflammatory and cytoprotective role is exerted by TAU-CL is exerted to protect macrophages and surrounding tissues from the toxic deleterious effect of overproduced reactive oxygen metabolites during inflammation [15]. Consistent with the antioxidant properties of TAU-CL observed in the present study, Zhang et al. (2014) [38] also found a protective role of TAU in hepatocytes subjected to iron overload as an inducer of oxidative stress.

In the present study, we decided to measure caspase-9, a protease upstream of caspase-3 and a downstream effector of the Bcl-2-mediated mitochondrial apoptosis pathway, indicating an increase in apoptotic activity in testicular tissue. Bcl-2 is a multidomain, prosurvival protein that regulates apoptosis by preventing the release of proapoptogenic factors from the mitochondria (e.g., cytochrome c) and subsequent caspase activation. In our study, AZA treatment for 4 weeks induced the activation of caspase-9 and significantly reduced levels of the anti-apoptotic protein Bcl-2. This dysregulation of the fine-tuned apoptosis pathway is considered one of the mechanisms of AZA-induced damage to testicular function and might be a reason for decreased sperm viability and mobility.

In agreement with our findings, Xu et al. (2016) [39] documented an increase in the activity of Bcl-2/Caspase-9 apoptosis pathway in the testis of asthmatic mice subjected to apoptotic inducers like ovalbumin. The protective role of TAU-CL was highlighted by the changes in caspase-9 activity and Bcl-2 levels toward the control values, which –based on our histopathological analysis – appears to have restored normal numbers of germ cells during spermatogenesis.

This putative anti-apoptotic effect of TAU-CL is supported by a report that suggests TAU inhibits apoptosis by preventing the formation of the Apaf-1/caspase-9 apoptosome [40]. In addition, Zulli et al. (2009) [41] reported an anti-apoptotic effect of TAU, which encourages the use of TAU as a dietary supplement in conditions where there are aberrant or inappropriate levels of apoptosis. A sensitive and indispensable method for revealing disturbances in spermatogenesis is histopathological examination. Our histological results revealed widening of the interstitial spaces and disruption and atrophy of many STs with reduced numbers of spermatogenic cells and spermatozoa in the AZA-treated group.

These results agree with those of Akinloye et al. (2011) [42], Nouri et al. (2009) [43] and Padmanabhan et al. (2009) [44], who reported that methotrexate caused significant increase in interstitial tissue and capsular thickness and decrease of testicular and body weight. Moreover, it caused significant decline in seminiferous tubule diameter and epithelium thickness, while cytoplasmic vacuolations were also reported by Karawya and El-Nahas (2006) [45].

The current study showed a decrease in the diameter of the STs, which was confirmed by morphometrical studies; this finding agrees with those of Shrestha et al. (2007) [46] and Khayatnour et al. (2011) [47] who reported that 60 days of methotrexate administration intraperitoneally (ip), decreased diameter of seminiferous tubules, increased interstial spaces as well as distortion of morphology of Leydig cells in experimental group. Many cells appeared shrunken with pyknotic nuclei and deeply acidophilic cytoplasm, which are hallmarks of apoptosis [48, 49]. AZA-induced reproductive dysfunction was confirmed by histomorphometrical analyses of rat testicular tissue; AZA significantly decreased the diameter and epithelial height of due to cell loss from the epithelium, epithelial sloughing in some tubules, and Leydig cell atrophy. Moreover, the results of this study showed a decrease in RI, TDI, and SPI, as presented in (Fig. 3b). As testosterone is essential for spermatogenesis, sperm maturation and motility any disruption in testosterone biosynthesis can adversely affect the structure and function of the testis [50].

Oral AZA administration in male rats caused a significant decrease in body and testicular weight and reduced serum testosterone, LH, and FSH levels, which we believe may be attributed to the oxidative stress [51] induced by this drug or decrease in the anabolic effects of testosterone [52, 53]. This hypothesis is consistent with the data reported by Duan et al. (2017) [54], who found a marked decline in serum FSH and LH levels and increased. In another study of El-Sharaky et al. (2010) [55], the use of a Leydig cell toxicant, Gossypol acetic acid (GAA), resulted in decreased testosterone levels in rats, resulting in increased germ cell apoptosis. In addition, testosterone can affect Sertoli cell function and germinal cell degeneration; thus, premature detachment of spermatids could occur due to Sertoli cell dysfunction and decreased testosterone levels, and low testosterone levels can enhance the premature detachment of epithelial cells [48, 56].

Leydig cell atrophy can be responsible for the reduction in serum testosterone levels. Therefore, changes in the seminiferous tubules, which were observed in the current histopathological study, may result from hormonal alterations induced by AZA and may not be a direct effect of the drug. Considering that normal spermatogenesis is adversely affected by increased oxidative stress and is promoted by increased endocrine activity in Leydig and Sertoli cells, it was not surprising that TAU was shown to protect spermatogenesis, decrease tubular atrophy and improve testicular and body weights [57]. This may be partly due to the reduction in oxidative stress indicators [17, 18], apoptotic markers and improved testosterone levels observed in this study [58].

Results of our study demonstrated significant increase of inducible nitric oxide synthase (iNOS) and nuclear factor-ƘB-p65 (NF-ƘB-p65) expression in AZA-treated group. In parallel to the present data Ilbey et al.(2009) [59] study has shown that cytotoxic chemotherapy species activate the nuclear factor-kB (NF-kB) family of transcription factors mediating inflammation and testicular damage.

The transcription factor NF-ƘB helps to control the expression of numerous genes activated during inflammation (i.e. cytokines, chemokines, growth factors, immune receptors, cellular ligands, inducible nitric oxide synthase [iNOS] and adhesion molecules). iNOS; is a protein that produces high amounts of nitric oxide (NO), and NO is highly reactive with other free radicals. Nitric oxide reacts with superoxide (O^−^) producing peroxynitrite, which in turn leads to protein nitration, DNA damage, and poly (ADP-ri- bose) polymerase activation. NF-ƘB can also be activated by oxidative stress [60].

Interestingly, we observed that TAU-CL-pretreatment exerted an inhibitory and downregulating effect on iNOS and NF-ƘB- p65 expression, thereby moderating the consequences of inflammation. In consistence with our findings, *Aydos* et al. *(2014)* [61] demonstrated the downregulating effect of taurine treatment on NO level and nitric oxide synthase expression in ischemia/reperfusion-induced testicular injury in a rat.

TAU-CL has shown to provide cytoprotection against AZA-induced testecular injury by inhibiting the overproduction of inflammatory mediators by the virtue of its anti-oxidative properties. As a direct antioxidant, taurine would quench and detoxify some reactive intermediates such as hypochlorous acid generated by myeloperoxidase. As an indirect antioxidant, taurine may protect cells via intercalating into the membrane and stabilizing it. Molecular gonadal protective effect of TAU was also documented by controlling the regulation of testicular-ERK1/2, -p38, -AKT and NF-ƘB [62], thereby moderating the consequences of inflammation.

Furthermore, the current study highlights that RT-PCR mRNA expression levels of Nrf2 and HO-1 were markedly decreased in AZA-treated group. Importantly, our results showed that pretreatment with taurine-chloramine significantly attenuated AZA-induced downregulation of Nrf2 and HO-1 expression. These findings are consistent with Yang et al. (2017) [63] who demonstrated the modulatory effect of taurine on Nrf2 and HO-1 expression levels against irradiation induced testicular damage in mice.

A plethora of studies suggest that in mammalian cells Nrf2 plays an important role to maintain normal cellular physiological conditions under exogenous oxidative insult by regulating the minimal and induced expression of several antioxidants molecules, enzymes including HO-1 and xenobiotic transporters [64], upregulation of HO-1expression through stimulation of Nrf2, conferring protection against oxidative damage and organ dysfunction [65]. Taurine-chloramine could therefore protect testes from Azathioprine (Imuran)-induced testicular damage and such process was relevant with Nrf2/HO-1 antioxidant pathway activation. These results were supported by the histopathological findings of a remarkably larger seminiferous tubules diameter and greater germinal epithelium height in testes from the TAU/AZA group compared to the AZA group.

Taurine-chloramine improved not only the morphological and histomorphometrical damage but also the apoptotic cell number and morphology which confirms the ability of TAU-CL to protect against the toxic effects of AZA in rats. Previous studies have reported that TAU treatment significantly prevented histomorphological damage and decreased the number of apoptotic cells in a rat model of diabetes-induced testicular dysfunction by suppressing the increase in oxidative stress [57, 59, 60].

As for the translation to medical care in oncologic diseases, the implication of TAU in clinical settings has proved effectiveness in reducing chemotherapy –induced toxicities. By virtue of its antioxidant impact, several studies pointed out that TAU co-administration decreases the risk of chemotherapy –induced hepatotoxicity, nephrotoxicity and also increases WBC count in patients with acute lymphocytic leukemia during their maintenance therapy [66]. It can also attenuate chemotherapy complications; e.g. nausea and vomiting, taste and smell impairment [67] as well as febrile episodes [68] and leads to a more tolerable chemotherapy with lower incidence of adverse drug events for the patients. In the field of chemotherapy supportive care, our results add to these reported benefits of TAU a promising protective effect against AZA induced male testicular atrophy, which can be experimented in a pilot study with a focus on testicular functions.

## Conclusion

We have characterized the destructive impact of AZA on the testes in male rats, and shown that TAU-CL supplementation can prevent AZA induced testicular inflammation, oxidative stress, and apoptosis, and can protect the histological and morphometrical features of normal testicular tissues. The benefit of TAU-CL in reducing AZA-induced oxidative stress and the inflammatory response, and hence testicular hypofunction, encourages its further investigation as a supplemental therapy when the use of AZA is mandatory. The chosen dose range and duration of AZA treatment in this study (5–10 mg/kg/day) resembled the clinically used doses and did not cause any drug interaction with TAU-CL or show altered efficacy. These findings warrant further studies to explore the exact molecular mechanism responsible for the protective effect of TAU or TAU-CL to establish their feasible application as a prophylactic therapy to chemotherapy treatment protocols.

## Abbreviations

6-MP: 6- mercaptopurine
AZA: azathioprine
BcL-2: B-cell lymphoma 2
CAT: catalase
DPA: diphenylamine
EH: epithelial height
GSH: glutathione
HO-1: heme oxygenase-1
IL-1β: interleukin-1 beta
iNOS: inducible nitric oxide synthase
MDA: malondialdehyde
NF-ƘB- p65: nuclear factor-ƘB-p65
NO: nitric oxide
Nrf2: The Nuclear factor erythroid2-related factor2
PAS: Periodic acid-Schiff reaction
RI: repopulation index
ROS: reactive oxygen species
SOD: superoxide dismutase
SPI: spermiogenesis index
STD: seminiferous tubule diameter
TAU: taurine
TAU-CL: taurine-chloramine
TCA: trichloroacetic acid
TDI: Tubular differentiation index
TNF-α: tumor necrosis factor-alpha
